# A Challenging Case of Kikuchi-Fujimoto Disease Associated with Systemic Lupus Erythematosus and Review of the Literature

**DOI:** 10.1155/2018/1791627

**Published:** 2018-01-23

**Authors:** Mihaela Găman, Ana-Maria Vlădăreanu, Camelia Dobrea, Minodora Onisâi, Cristina Marinescu, Irina Voican, Daniela Vasile, Horia Bumbea, Diana Cîşleanu

**Affiliations:** ^1^Carol Davila University of Medicine and Pharmacy, Bucharest, Romania; ^2^Department of Hematology, University Emergency Hospital Bucharest, Bucharest, Romania

## Abstract

Kikuchi–Fujimoto disease (KFD) or histiocytic necrotizing lymphadenitis is a rare disease that is frequently underdiagnosed due to clinical features that are similar to those of non-Hodgkin lymphomas, systemic lupus erythematosus (SLE), or infectious reactive lymphadenopathy. An excisional biopsy is required. We report a young Caucasian female diagnosed with KFD with skin lesions, complicating with SLE. The clinical course, laboratory, and CT findings are described, as are histopathologic features, for a better recognition of this rare disorder in clinical practice.

## 1. Introduction

Kikuchi–Fujimoto disease (KFD) is a self-limiting disease that is characterized by cervical adenopathies and fever [1, 2]. Although KFD is reported in various ethnic groups and geographical areas, young Asian females are frequently affected. The onset is acute or subacute, with painful cervical nodes and fever in previously young individuals who were previously healthy. Extranodal manifestations are rare [3]. The skin is commonly affected (16–40% of patients), with nonspecific lesions [4]. The diagnosis represents a challenge due to similar features to non-Hodgkin lymphoma (NHL), systemic lupus erythematosus (SLE), and reactive lymphadenopathy; however, examination by an experienced morphopathologist offers a correct diagnosis. KFD lymphadenitis presents coagulative necrosis and karyorrhectic debris [3].

Several reports have emphasized the importance of KFD and SLE association. KFD can precede or coexist with SLE [4]. Some authors recommend antinuclear antibody (ANA) screening at diagnosis and close follow-up, especially in patients with cutaneous lesions for the early detection of an autoimmune disease [5–8].

## 2. Case Report

A 26-year-old Caucasian female was admitted to the hospital with fever, chills, and malaise. The patient reported fever of up to 39°C for the past 4 weeks, intermittent arthralgia of bilateral proximal and distal interphalangeal joints of the hands. One year prior to this, she presented with a similar episode of fever and tender cervical lymph node. She underwent a biopsy with a diagnosis of reactive lymphadenitis. The symptoms resolved spontaneously. No tests to exclude an autoimmune disease were performed. Her medical history included insulin-dependent type 1 diabetes.

Clinical examination revealed a febrile patient with enlarged and tender bilateral cervical and axillary lymph nodes as well as hepatomegaly. Scattered erythematous macules were noted on the neck, trunk, and upper extremities.

Laboratory studies are presented in Table 1. She had a high erythrocyte sedimentation rate, moderate anemia, leukopenia with marked lymphopenia, negative inflammatory markers, increased hepatic enzymes, hypoalbuminemia, and albuminuria (1815 mg/24 h). Blood cultures were negative. Serologic tests for hepatitis B and C, HIV, Epstein–Barr virus, cytomegalovirus, and toxoplasmosis were negative. She had a positive ANA titer (1/3200) and positive double-strand DNA antibodies (2628 UI/mL), negative rheumatoid factor, C3 complement, and smooth muscle antibodies. Normocellular marrow with no tumor cells was found on bone marrow biopsy.

CT scan revealed generalized lymphadenopathy involving the submandibular, cervical, supraclavicular, and axillary areas, the mediastinum, and the intra-abdominal and inguinal regions ranging in size from 0.9 cm to 2.5 cm. CT also revealed hepatomegaly (right lobe 21 cm craniocaudal diameter) and pleural effusion (2.3 cm right side and 0.5 cm left side).

**Figure 1:**
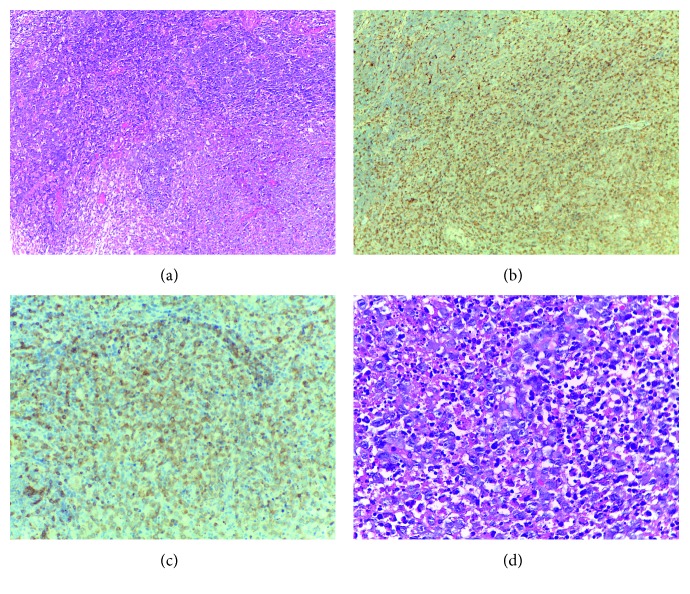
(a) H&E stained section (10x) of lymph node. Foci of necrosis containing abundant karyorrhectic debris. (b) Immunohistochemical stained section (10x). CD68-positive histiocytes. (c) Immunohistochemical stained section (10x). Abundant CD3 dim positive immunoblastic T-cells. (d) H&E stained section (40x) of lymph node. Infiltration pattern with abundant transformed lymphocytes with immunoblast morphology.

Excisional biopsy of axillary lymphadenopathy was diagnostic for KFD (Figure 1). There was an effaced architecture with foci of necrosis containing karyorrhectic debris, a polymorphic infiltration pattern with abundant histiocytes, large transformed lymphocytes with immunoblast morphology, and rare tangible macrophages. No polymorphonuclear leukocytes or plasma cells were identified. Immunohistochemical staining showed abundant CD3 dim positive immunoblastic activated cytotoxic T-cells that outnumbered CD20 immunoblastic B-cells, scattered CD56 NK cells, rare CD30 cells, and a large number of CD68/KP1 histiocytes. Pankeratin and ALK were negative.

Antipyretics and nonsteroidal anti-inflammatory drugs were started with slow clinical improvement. The patient was treated with prednisone 1 mg/kg/day × 2 weeks, which was then tapered. The arthralgias improved, fever did not reappear, and a gradual attenuation of skin lesions was noted. Soon after hospitalization in the Hematology Department, she was referred to the rheumatologist for evaluation and closer follow-up of her autoimmune disease.

## 3. Discussion

Kikuchi disease occurs in a wide age range of patients but usually affects young adults. The age of diagnosis in our patient was similar to that in other published series (median age of 20 to 30 years), including mostly Asian females with a male : female ratio of 1 : 2 [9–11]. Kikuchi disease may be underdiagnosed due to similarities with other disorders. Recent data reported increasing incidence outside of Asia for all races [12–14], yet very rare cases of KFD are reported in Caucasian population and the exact incidence is unknown.

Pathogenesis remains unclear. Various infections have been proposed, such as Epstein–Barr virus, human herpes virus, herpes simplex virus, hepatitis B, parvovirus B19, human T-lymphotrophic virus 1, *Yersinia enterocolitica*, and toxoplasma [4, 15]. This theory is based on the self-limited course, laboratory findings (atypical morphologic lymphocytes on peripheral blood smear, as well as elevated inflammatory cytokines interferon *α*, and interleukin 6), and histological features (CD8 + T-lymphocytes that induce cell apoptosis). These cells later undergo apoptosis themselves, resulting in a necrosis background [3, 16, 17]. However, the results are inconclusive, and it is not clear whether infections are responsible for pathogenesis. Other reports suggested an autoimmune origin because KFD has been reported to simultaneously occur or follow SLE. Our patient also associated with autoimmune features—SLE criteria in addition to a 17-year-history of diabetes type 1. Other autoimmune diseases (Still's disease, Sjogren's syndrome, polymyositis, and rheumatoid arthritis) have rarely been reported to be associated with KFD [4, 18–20].

Lymphadenopathies are the most relevant manifestations. The cervical area is a frequently involved site with severe pain or tenderness. KFD usually presents as solitary or multiple enlarged cervical lymph nodes and rarely with generalized lymphadenopathies, such as in our case [21–23].

Fever is another manifestation, which is present in 30–50% of reported cases. Less common clinical findings include weight loss, malaise, chills, night sweats, or gastrointestinal symptoms [24].

Few reports noted hepatomegaly as presenting extranodular sites. Biopsies performed in published cases revealed that reactive changes and hepatic enzymes reverted to normal after a short period of time, as in our patient [11].

Nonspecific cutaneous manifestations (such as erythematous macules, papules, plaques, facial malar erythema, erosions, patches, and nodules) have been observed in up to 40% of cases [25]. In our case, skin lesions preceded the appearance of adenopathies and resolved 3-4 weeks after initiating treatment. Unfortunately, a skin biopsy was not performed. It would have been of great value to distinguish KFD from SLE skin lesions, and we strongly recommend it to be performed whenever possible. Histological findings such as perivascular and interstitial infiltration of CD68- and CD163-positive histiocytes, CD8-positive cytotoxic T-lymphocytes, nuclear debris, the absence of neutrophils, vacuolar degeneration, keratinocyte necrosis, and the presence of interface dermatitis have been described in KFD cutaneous lesions [26, 27]. The KFD cutaneous lesions could resemble clinically and morphologically with skin manifestations observed in SLE: the presence of interface dermatitis, dermal mucin deposition, and panniculitis. The difference is in the absence of plasma cells that are commonly found in SLE [26].

Biological findings such as leukopenia, neutropenia, atypical lymphocytes on peripheral blood smear, thrombocytopenia, and anemia have been classically reported in KFD. It is notable that our patient displayed lymphopenia which is not commonly seen in KFD and has rather been reported when SLE associated with KFD [18, 28]. Reports also associate severe lymphopenia with more forms of KFD [28].

In addition to these, other biological abnormalities have also been reported: elevated inflammatory markers, elevated liver transaminases, increased LDH, ANA, and reduced C3 values [11, 29].

Our patient fulfilled SLE diagnosis criteria according to both ACR and SLICC classification [30], respectively (a total of 6 criteria): serositis (pleural effusion), arthritis (tenderness in bilateral proximal and distal interphalangeal joints of hands), proteinuria greater than 500 mg/24 hours (1815 mg), leukopenia (2200/mmc) with significant lymphopenia (approx. 300/mmc), double-stranded DNA, and ANA positivity (immunologic criteria). Sopeña et al. reported an incidence of 23% SLE cases in 20 patients with Kikuchi disease and autoimmune manifestations. The incidence was similar to those in previous literature [18]. According to Kucukardali et al., SLE-associated Kikuchi's disease is more common in Asian patients than in European patients. 28 patients were studied: 18 had simultaneous SLE and KFD, 6 presented with SLE after KFD diagnosis, and 4 were previously diagnosed with SLE [11]. The association between these two disorders is not completely understood. The clinical features are similar, and differentiation between them is based on lymph node histopathology [31]. The absence of hematoxylin bodies and neutrophils indicates KFD rather than SLE, and our patient showed definite features of Kikuchi's disease. Histopathologic features that support SLE include an increased number of plasma cells, hematoxyphilic bodies, DNA deposits in the vascular walls, neutrophilic infiltration, and varying degrees of coagulative necrosis with Azzopardi phenomenon [3]. Given the number of published clinical cases, the association between KFD and SLE cannot be random. Some authors have postulated that KFD is prodromal for SLE and recommend follow-up for KFD patients with cutaneous lesions for detecting an autoimmune disease. Others recommend ANA screening at the time of KFD diagnosis [7, 19, 27].

Differentiating KFD from other diseases such as lymphoma and reactive lymphadenopathies (e.g., sarcoidosis, tuberculosis, toxoplasmosis, and cat-scratch fever) is mandatory [22, 32]. Our patient was referred to the hematologist with the clinical suspicion of NHL, particularly angioimmunoblastic T-cell lymphoma, as it may associate with rash, arthralgias, and ANA positivity [33]. The differential diagnosis of the two most clinical relevant findings, fever and lymphadenopathy, is often challenging and requires an extensive work-up. This is emphasized in a group of 244 KFD patients published by Kucukardali et al. [11]. Our patient had a normal bone marrow examination and negative virology serological tests.

Even with adequate biopsy, appearances can be mistaken for NHL or reactive lymphadenopathies [22]. In our patient, an experienced morphopathologist confirmed KFD on the second biopsy. Coagulative necrosis with histiocytes proliferation and the absence of granulocytic infiltration is mandatory for diagnosis. Necrosis, karyorrhexis, and cellular debris are the hallmarks of the necrotizing type. Kuo proposed three histopathological stages: (a) a *proliferative stage* with histiocytes, plasmacytoid monocytes, lymphoid cells, karyorrhectic nuclear fragments, and eosinophilic apoptotic debris; (b) a *necrotizing stage* with coagulative necrosis; and (c) a *xanthomatous stage* with foamy histiocytes predominance [3]. In our case, the biopsy confirmed the necrotizing stage. A helpful histopathological feature is the absence of granulocytes in the necrotizing stage, which is used to distinguish KFD from SLE or reactive lymphadenopathy, especially given the coexistence of clinical or biological abnormalities that are similar to those of an autoimmune disease.

*Recurrence* is rarely reported (3–5%). Song et al. reported a higher incidence rate in ANA-positive cases and identified fever, fatigue, extranodal involvement, and positive fluorescence antinuclear antibody as predictive factors for relapse [34]. Our patient had presented a previous episode of fever and enlarged lymph nodes. However, the excisional biopsy did not suggest KFD and unfortunately was not available for reexamination. That is why we emphasize again the need for an expert morphopathologic evaluation in any case with features resembling KFD.

The *outcome* of these patients is generally favorable with a self-limited course. *Treatment* is symptomatic with NSAIDs and a short course of steroids. KFD coexisting with SLE can have a more aggressive course, and treatment is recommended to prevent relapse, as in our case. Rare cases with a fatal evolution have been reported; KFD was found to be complicated with hemophagocytic syndrome, heart failure, or recurrent aseptic meningitis that required intravenous immunoglobulin and corticosteroids [35–38].

## 4. Conclusions

Many of the symptoms, cutaneous manifestations, biological and histopathological features of KFD are similar to those of autoimmune diseases, particularly those of SLE, and differentiating between the two entities is challenging.

We present this case in order to highlight the rare clinical entity of Kikuchi's disease in Caucasian population, the need for an experienced pathologist and for long-term follow-up, especially for those cases with cutaneous manifestations, and high risk of associating autoimmune diseases such as SLE.

## Figures and Tables

**Table 1:** Laboratory tests.

Parameter	Results	Normal range
ESR (mm/h)	68	<20
Hemoglobin (g/dl)	8.7	14–18
Hematocrit (%)	27.0	42–52
WBC (×10^9^/mmc)	2.2	4–10
Differential	80% neutrophils, 14% lymphocytes, 2% bands, 4% monocytes	
PLT (×10^9^/mmc)	238	150–450
CRP (mg/l)	3.88	<5
Procalcitonin (ng/mL)	<0.5	<0.5
AST (U/L)	89	2–40
ALT (U/L)	120	3–65
GGT (U/L)	190	5–85
LDH (U/L)	345	125–220
Albumin (g/dl)	1.9	3.4–5.2
Albuminuria (mg/dl)	72.6	0–3
Albuminuria/24 hours	1815 mg/24 h	
